# Prognostic utility of serum albumin in underweight patients who underwent isolated surgical aortic valve replacement

**DOI:** 10.1007/s11748-026-02299-3

**Published:** 2026-04-27

**Authors:** Takahiro Ohmori, Koichi Maeda, Kyongsun Pak, Akinobu Ohtani, Yusuke Yanagino, Kazuma Handa, Takura Taguchi, Koichi Inoue, Ai Kawamura, Shigeru Miyagawa

**Affiliations:** 1 Department of Cardiovascular Surgery, The University of Osaka, 2-2 Yamadaoka, Suita, Osaka 565-0871 Japan; 2 Division of Biostatistics, Center for Clinical Research, National Center for Child Health and Development, 2-10-1 Okura, Setagaya-ku, 157-8535 Tokyo, Japan

**Keywords:** Body mass index, Albumin, Aortic stenosis, Surgical aortic valve replacement

## Abstract

**Objective:**

The clinical impact of underweight on outcomes in patients undergoing surgical aortic valve replacement (SAVR) remains unclear. This study aimed to investigate the prognostic significance of underweight status in patients undergoing SAVR and to identify key determinants of adverse outcomes within this population.

**Methods:**

805 patients underwent SAVR between January 2016 and December 2021 at eight Japanese centers. After excluding 31 patients with missing laboratory data, a total of 774 patients were included. Patients were categorized into underweight group (Body mass index [BMI] < 18.5 kg/m², 79 patients), normal-weight group (BMI 18.5–25 kg/m², 464 patients), and overweight group (BMI　≥25 kg/m², 231 patients). The primary endpoint was all-cause mortality.

**Results:**

The median age was 76 years (interquartile range [IQR]: 71–79), and the median BMI was 23.3 kg/m² (IQR: 20.9–25.8). Underweight patients had the highest 5-year mortality, followed by normal-weight and overweight patients (23.5%, 11.9%, and 7.4%; *p* = 0.001). In multivariable analysis, BMI was an independent predictor of mortality (1.0 kg/m² increase, adjusted hazard ratio: 0.92; 95% confidence interval: 0.86 to 0.99; *p* = 0.020). Among underweight patients, serum albumin was independently associated with all-cause mortality (*p* = 0.002). Underweight patients were stratified by median albumin into low (< 4.0 g/dL, *n* = 43) and normal (≥ 4.0 g/dL, *n* = 36) groups, and 5-year mortality was higher in the low albumin group (34.1% vs. 12.6%, *p* = 0.015).

**Conclusions:**

Low BMI was associated with higher mortality after SAVR, and serum albumin provided additional risk stratification within underweight group.

**Supplementary Information:**

The online version contains supplementary material available at https://doi.org/10.1007/s11748-026-02299-3.

## Introduction

Underweight (body mass index [BMI] < 18.5 kg/m²) is less common in Western countries, and its prognostic impact after surgical aortic valve replacement (SAVR) has been insufficiently studied. In addition, because the patient profile of underweight individuals may differ in Asia, the clinical significance of underweight in Asian SAVR patients may not be directly inferred from Western data. In patients with severe aortic stenosis (AS), accurate preoperative risk assessment is essential for selecting the most appropriate treatment strategy, [1, 2] as SAVR and transcatheter aortic valve replacement (TAVR) are currently established as the two main therapeutic options. The Society of Thoracic Surgeons Predicted Risk of Mortality (STS-PROM) score is widely applied for operative risk assessment [3, 4]. However, because the STS-PROM model was developed with a valid BMI input range of 18–50 kg/m², BMI values < 18 kg/m² are truncated to 18 kg/m². Consequently, patients with BMI values below this threshold cannot be accurately stratified, and their true operative risk remains uncertain. Among underweight patients, some are frail or malnourished, whereas others are constitutionally small but otherwise healthy, leading to heterogeneous clinical outcomes [5]. Accordingly, the present study aimed to examine the prognostic impact of underweight in patients undergoing SAVR and to identify clinical determinants of outcome within the underweight population.

## Materials and methods

### Study population

The ACTIVIST registry is a multicenter, retrospective registry that enrolled patients with severe AS who underwent either primary isolated SAVR or TAVR between February 2016 and December 2021 across eight centers in Japan. Patient characteristics, clinical risk factors and comorbidities, echocardiographic data, procedural details, and follow-up outcomes were retrospectively collected in a centralized database. Echocardiographic data were collected before surgery and at discharge. The study protocol was approved by the Institutional Review Board or ethics committee of each participating center. Severe AS was defined as an aortic valve area < 1.0 cm², an indexed aortic valve area < 0.6 cm²/m², a mean transvalvular pressure gradient ≥ 40 mmHg, or a peak aortic jet velocity ≥ 4.0 m/s. The registry initially included 2,811 patients. For the present study, 805 patients who underwent isolated SAVR were selected from this cohort. The study was conducted in accordance with the principles of the Declaration of Helsinki and was approved by the Clinical Research Ethics Committee of Osaka University Hospital (Approval No.: 20222 [T2]; Approval Date: December 15, 2021).

### Body mass index and albumin classification

**Fig. 1:**
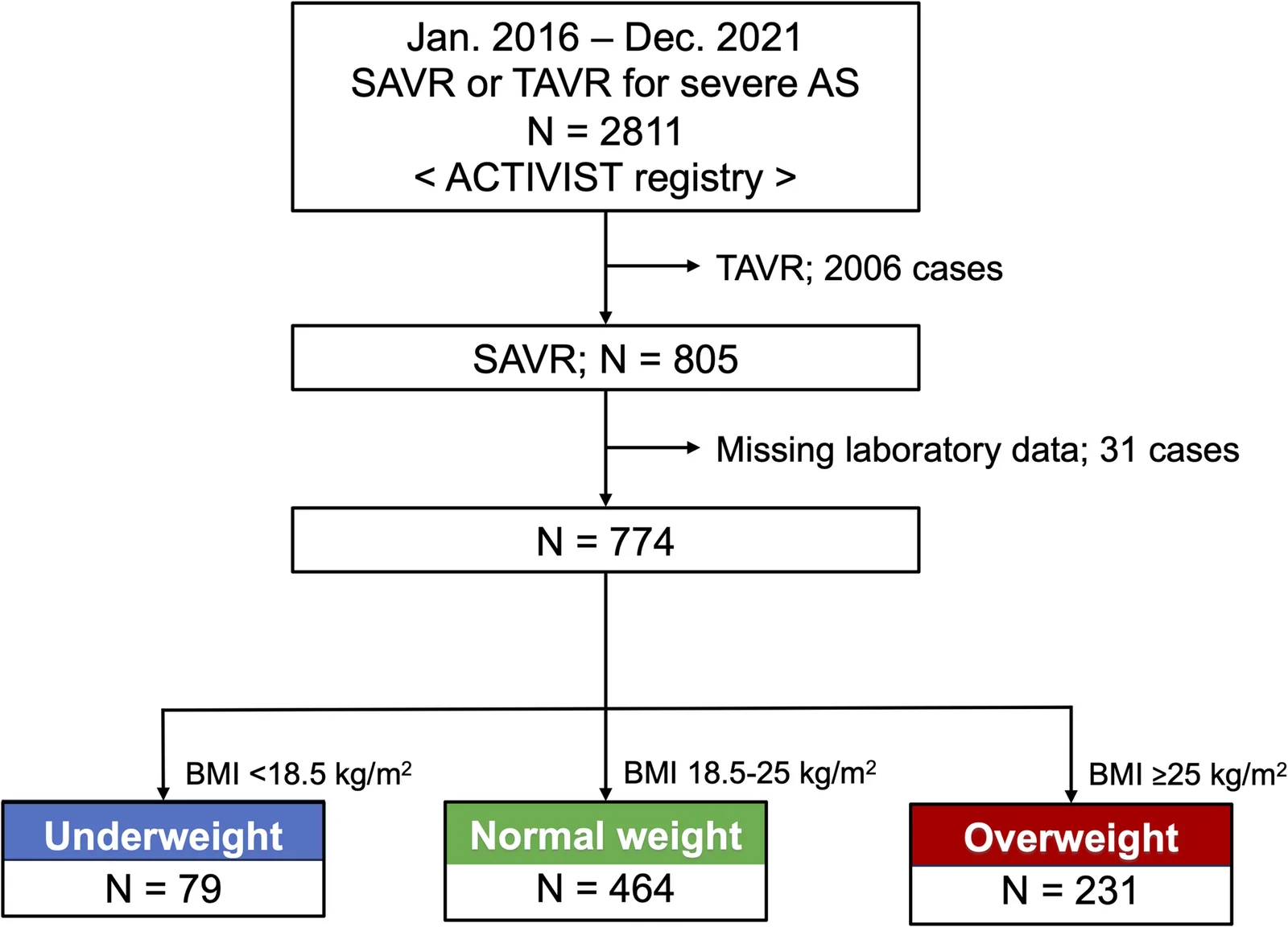
Flowchart depicting the selection of the study population

Patients were classified according to the World Health Organization BMI criteria: underweight (< 18.5 kg/m²), normal weight (18.5–25 kg/m²), and overweight/obese (≥ 25 kg/m²). Patients with missing laboratory data (*n* = 31) were excluded from the analysis. Consequently, a total of 774 patients were included in the final analysis. 79 patients were categorized as “underweight,” 464 patients as “normal weight,” and 231 patients as “overweight.” (Fig. 1).

### Endpoints

The primary study endpoint was all-cause death at five years. The secondary endpoints included cardiovascular death and non-cardiovascular death at five years. Both cardiovascular and non-cardiovascular deaths were defined according to the Valve Academic Research Consortium 3 criteria [6].

### Statistical analysis

Continuous variables are presented as a median (interquartile range [IQR]), and categorical variables are expressed as counts and percentages. Between-group differences were assessed using one-way analysis of variance or the Kruskal–Wallis test for continuous variables, and the χ2 test or Fisher’s exact test for categorical variables, as appropriate. The cumulative incidence of each endpoint was analyzed using the Kaplan–Meier model, and the differences among the groups were assessed with the log-rank test. Predictors of the primary endpoint were evaluated by Cox proportional hazard regression models. Variables with a *p*-value < 0.05 in the univariable analysis and those clinically relevant based on previous studies were included in the multivariable analysis. A restricted cubic spline analysis was performed to explore the potential non-linear association between BMI and all-cause mortality in the entire cohort, using the median BMI as the reference. In addition, the same approach was used to examine the association between serum albumin level and all-cause mortality among underweight patients, with 4.0 g/dL as the reference. All data were analyzed using the Statistical Analysis Systems software JMP 18.0 (SAS Institute Inc., Cary, NC, USA) and R software version 4.3.0 (R Foundation for Statistical Computing, Vienna, Austria).

## Results

The median age was 76 years (IQR: 71–79), and 52.8% of the patients were female. The median BMI was 23.3 kg/m² (IQR: 20.9–25.8). Patient characteristics are summarized in Table 1. Among the three BMI groups, underweight patients were older and had higher STS-PROM scores, and the prevalence of hypertension, diabetes mellitus, and dyslipidemia was lower in the underweight group. Postoperative echocardiographic findings are shown in Table 2. Among the three BMI groups, there were no significant differences in the incidence of prosthesis–patient mismatch or paravalvular leak.

**Table 1:** Baseline patients’ characteristics

	Underweight	Normal weight	Overweight	*p* value
	*N* = 79	*N* = 464	*N* = 231	
**Clinical data**				
Age, year	77 [71–82]	76.00 [71–80]	74 [69–78]	**0.002**
Female	49 (62.0)	228 (49.1)	132 (57.1)	**0.031**
Body surface area, m^2^	1.38 [1.29–1.49]	1.53 [1.42–1.65]	1.67 [1.56–1.83]	**< 0.001**
Body mass index, kg/m^2^	17.0 [15.7–18.0]	22.4 [20.9–23.7]	27.7 [26.2–29.7]	**< 0.001**
STS score, %	3.36 [1.92–5.08]	2.95 [1.83–4.82]	2.12 [1.23–3.22]	**< 0.001**
NYHA class Ⅲ or Ⅳ	17 (21.5)	92 (19.8)	36 (15.6)	0.32
Hypertension	50 (63.3)	321 (69.2)	188 (81.4)	**< 0.001**
Diabetes	14 (17.7)	125 (26.9)	84 (36.4)	**0.003**
Hyperlipidemia	31 (39.2)	243 (52.4)	147 (63.6)	**< 0.001**
Stroke	4 (5.1)	44 (9.5)	16 (6.9)	0.28
Smoking	20 (25.3)	99 (21.3)	52 (22.5)	0.72
Coronary artery disease	4 (5.1)	58 (12.5)	30 (13.0)	0.14
Atrial fibrillation	10 (12.7)	79 (17.0)	22 (9.5)	**0.026**
Pacemaker implantation	3 (3.8)	16 (3.4)	2 (0.9)	0.13
Chronic lung disease ≥moderate	5 (6.3)	21 (4.6)	5 (2.2)	0.18
Chronic kidney disease	43 (54.4)	269 (58.0)	120 (51.9)	0.31
eGFR, mL/min/1.73 m^2^	58.4 [40.50–73.4]	56.4 [41.6–71.4]	59.3 [49.1–70.1]	0.12
Albumin, g/dL	3.90 [3.60–4.20]	4.00 [3.70–4.20]	4.00 [3.80–4.30]	**0.001**
**Ankle Brachial Index**				
Right	1.09 [1.03–1.16]	1.10 [1.02–1.16]	1.09 [1.04–1.16]	0.85
Left	1.09 [1.04–1.15]	1.08 [1.01–1.15]	1.09 [1.04–1.16]	0.31
**Echocardiographic data**				
LVEF, %	66 [56–74]	67 [59–73]	68 [61–72]	0.66
AVA, cm^2^	0.64 [0.50–0.77]	0.72 [0.59–0.85]	0.79 [0.65–0.89]	**< 0.001**
AVAi, cm^2^/m^2^	0.46 [0.37–0.57]	0.47 [0.38–0.55]	0.46 [0.40–0.53]	0.77
MPG, mmHg	52 [41–63]	49 [40–61]	48 [41–57]	0.55
Peak velocity, m/s	4.68 [4.22–5.20]	4.60 [4.20–5.10]	4.55 [4.20–4.96]	0.27
TRPG, mmHg	26 [19–34]	25 [21–30]	23 [19–29]	0.10
LVM, g	187 [151–228]	194 [159–236]	208 [174–249]	**< 0.001**
LVMi, g/m^2^	128.5 [107.6-158.9]	126.1 [103.5-151.9]	124.9 [106.3-143.6]	0.38
E/A	0.76 [0.59–0.91]	0.71 [0.58–0.88]	0.67 [0.55–0.82]	0.060
E/e’	16.3 [12.0-23.3]	15.0 [11.8–21.0]	16.2 [12.4–21.0]	0.69
moderate to severe AR	20 (25.3)	66 (14.2)	32 (13.9)	**0.031**
moderate to severe MR	8 (10.1)	34 (7.3)	13 (5.6)	0.39
moderate to severe TR	6 (7.6)	13 (2.8)	5 (2.2)	**0.048**
Bicuspid valve	18 (22.8)	111 (23.9)	50 (21.6)	0.80
**Procedural data**				
Valve size ≤ 21 mm	54 (68.4)	279 (60.1)	126 (54.5)	0.083
Valve type				
Bioprosthetic valve	73 (92.4)	432 (93.1)	207 (89.6)	0.28
Mechanical valve	6 (7.6)	32 (6.9)	24 (10.4)	
Clamp time, min.	92 [70–107]	96 [79–115]	97 [76–118]	0.076
Pump time, min.	128 [103–157]	135 [113–164]	139 [112–168]	0.083

Values are n (%), or median [interquartile range]. Bold indicates *p* < 0.05.

AR, aortic regurgitation; AVA, aortic valve area; AVAi, aortic valve area indexed to body surface area; eGFR, estimated glomerular filtration rate; LVEF, left ventricular ejection fraction; LVM, left ventricular mass; LVMi, left ventricular mass indexed to body surface area; MPG, mean pressure gradient; MR, mitral regurgitation; NYHA, New York Heart Association; STS, Society of Thoracic Surgeons; TR, tricuspid regurgitation; TRPG, tricuspid regurgitation pressure gradient.

**Table 2:** Postoperative echocardiographic data and 30-day clinical outcomes

	Underweight	Normal weight	Overweight	*p* value
	*N* = 79	*N* = 464	*N* = 231	
**Echocardiographic data**				
LVEF, %	63 [54–69]	63 [56–68]	62 [58–68]	0.94
MPG, mmHg	12 [9–15]	12 [9–15]	12 [9–15]	0.44
EOA, cm^2^	1.39 [1.21–1.58]	1.47 [1.25–1.74]	1.55 [1.30–1.84]	**0.004**
EOAi, cm^2^/m^2^	0.99 [0.85–1.16]	0.96 [0.81–1.14]	0.92 [0.77–1.07]	**0.031**
PPM	16 (25.0)	119 (31.6)	68 (37.4)	0.15
Moderate PPM	11 (17.2)	108 (28.6)	58 (31.9)	
Severe PPM	5 (7.8)	11 (2.9)	10 (5.5)	
moderate to severe PVL	1 (1.3)	1 (0.2)	0 (0.0)	0.28
moderate to severe MR	0 (0.0)	13 (2.8)	1 (0.4)	**0.035**
moderate to severe TR	3 (3.9)	11 (2.4)	1 (0.4)	0.12
**30-day clinical outcomes**				
All-cause death	1 (1.3)	3 (0.7)	0 (0.0)	0.33
Cardiovascular death	0 (0.0)	1 (0.2)	0 (0.0)	0.72
Stroke	0 (0.0)	8 (1.7)	4 (1.7)	0.50
Permanent pacemaker implantation	1 (1.3)	9 (1.9)	6 (2.6)	0.74
Heart failure rehospitalization	0 (0.0)	2 (0.4)	1 (0.4)	0.84
Reintervention	0 (0.0)	0 (0.0)	0 (0.0)	N/A

Values are n (%), or median [interquartile range]. Bold indicates *p* < 0.05

LVEF, left ventricular ejection fraction; MPG, mean pressure gradient; N/A, not applicable; EOA, effective orifice area; EOAi, effective orifice area indexed to body surface area; PPM, prosthesis–patient mismatch; PVL, paravalvular leak; MR, mitral regurgitation; TR, tricuspid regurgitation

### Relation between body mass index and clinical outcomes

**Fig. 2:**
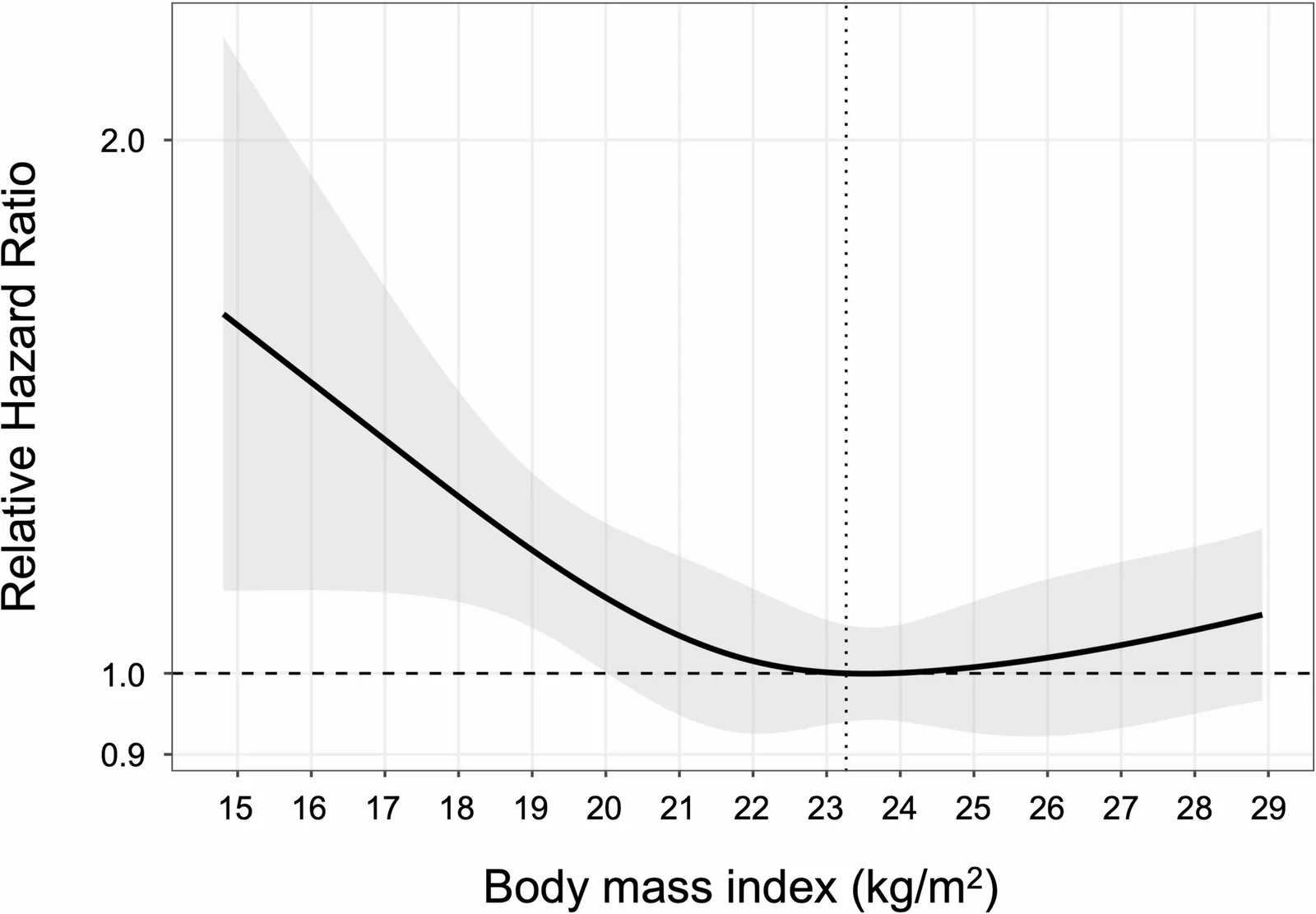
Restricted cubic spline analysis illustrating the continuous association between body mass index and all-cause mortality. Hazard ratios (solid line) and 95% confidence intervals (shaded area) are shown. The reference body mass index was set at the median value of 23.3 kg/m²

Restricted cubic spline analysis showed that the hazard ratio for all-cause mortality increased as BMI decreased below the median BMI of 23.3 kg/m² (Fig. 2). In multivariable Cox proportional hazards analyses, BMI as a continuous variable was significantly associated with all-cause mortality (adjusted hazard ratio [HR]: 0.92; 95% confidence interval [CI]: 0.86 to 0.99; *p* = 0.020). When BMI was categorized into three groups, underweight was associated with higher all-cause mortality compared with normal weight (adjusted HR: 2.42; 95% CI: 1.25 to 4.69; *p* = 0.016), whereas overweight was not significantly associated with mortality (adjusted HR: 0.91; 95% CI: 0.47 to 1.73; *p* = 0.77) (Table 3).

**Table 3:** Association between body mass index and all-cause mortality in multivariable Cox proportional hazards analysis

	Univariable		Multivariable (Model 1)		Multivariable (Model 2)	
	HR (95% CI)	*p* value	Adjusted HR (95% CI)	*p* value	Adjusted HR (95% CI)	*p* value
BMI, per 1.0 kg/m2	0.88 (0.83–0.94)	**< 0.001**	0.92 (0.86–0.99)	**0.020**		
BMI category						
Normal weight	1.00 (Reference)				1.00 (Reference)	
Underweight	2.11 (1.16–3.85)	**0.023**			2.42 (1.25–4.69)	**0.016**
Overweight	0.56 (0.30–1.03)	0.052			0.91 (0.47–1.73)	0.77

Multivariable analyses were adjusted for age, sex, Society of Thoracic Surgeons score, coronary artery disease, chronic kidney disease, moderate to severe tricuspid regurgitation, and albumin. Model 1; BMI is included in the model as a continuous variable. Model 2; BMI is included in the model as a categorical variable.

Bold indicates *p* < 0.05. BMI, body mass index; CI, confidence interval; HR, hazard ratio.

## Underweight and clinical outcomes

**Fig. 3:**
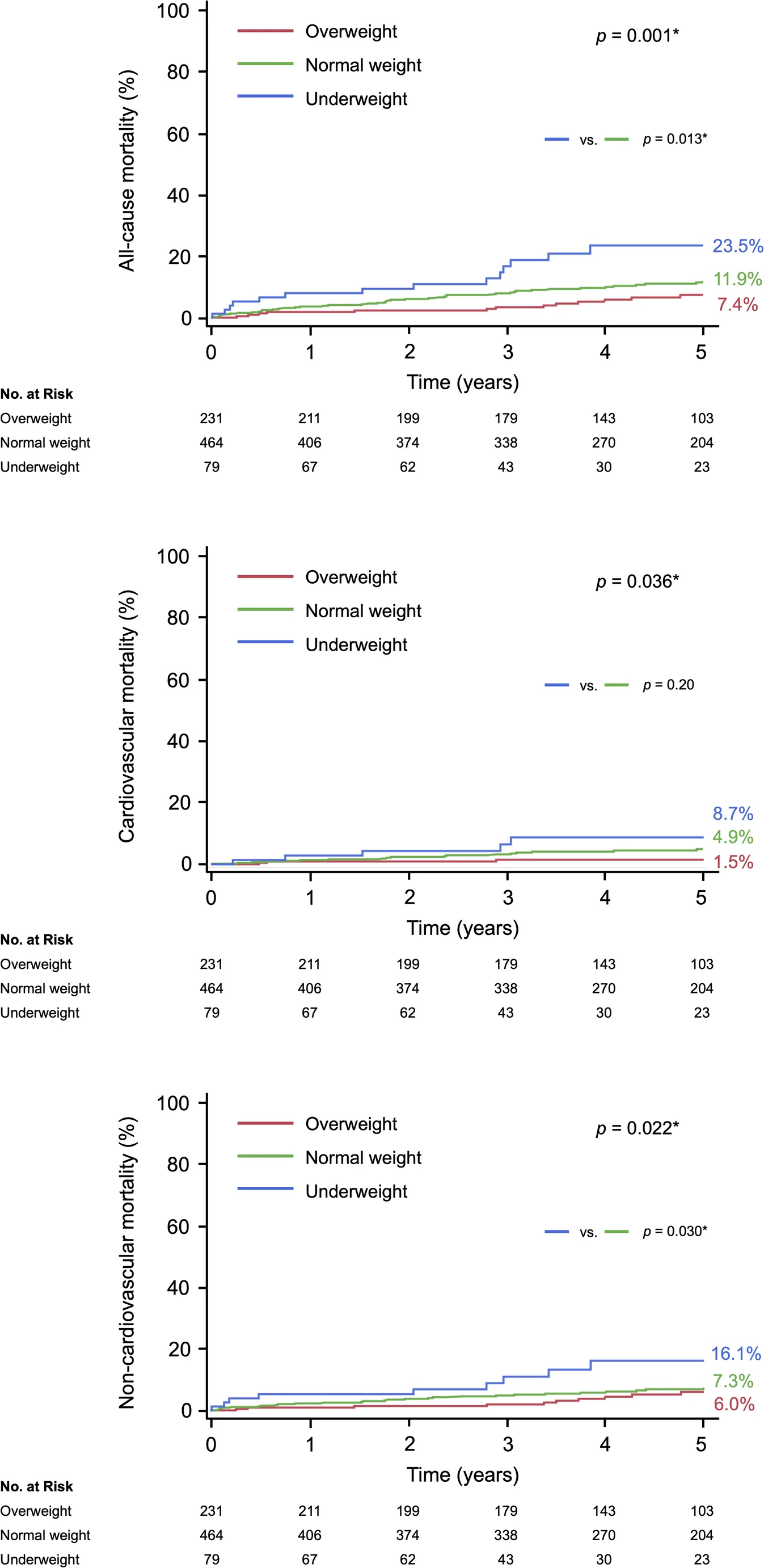
Kaplan–Meier survival curves comparing normal-weight and underweight patients. (**A**) All-cause mortality, (**B**) non-cardiovascular mortality, and (**C**) cardiovascular mortality

Thirty-day clinical outcomes according to BMI category are summarized in Table 2. The 5-year incidence of all-cause mortality differed significantly across BMI categories, with the highest incidence observed in underweight patients, followed by normal-weight and overweight patients (23.5%, 11.9%, and 7.4%, respectively; log-rank *p* = 0.001; Fig. 3A). A similar pattern was observed for cardiovascular death, which was highest in underweight patients (8.7%, 4.9%, and 1.5%, respectively; *p* = 0.036; Fig. 3B), and for non-cardiovascular death (16.1%, 7.3%, and 6.0%, respectively; *p* = 0.022; Fig. 3C). In a comparison between underweight and normal-weight patients, all-cause mortality (*p* = 0.013) and non-cardiovascular death (*p* = 0.030) were significantly higher in underweight patients, whereas cardiovascular death was not significantly different (*p* = 0.20).

The distribution of causes of non-cardiovascular death is summarized in Supplemental Figure E1. Although the distribution of causes did not differ significantly between groups (*p* = 0.19), infection-related death was frequent among underweight patients (87.5%).

## Stratification of underweight patients by serum albumin level

**Fig. 4:**
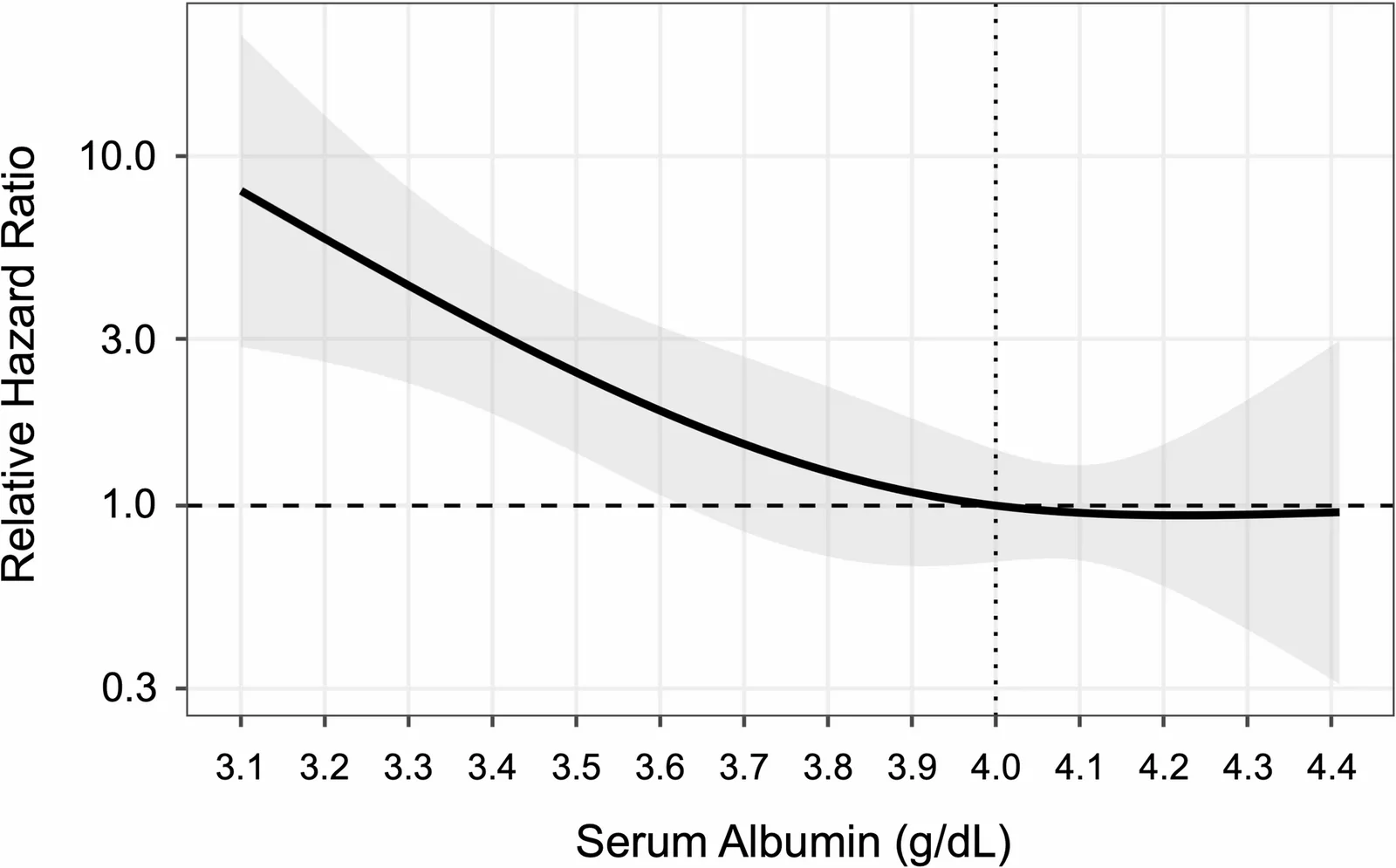
Restricted cubic spline analysis illustrating the continuous association between serum albumin level and all-cause mortality in underweight patients. Hazard ratios (solid line) and 95% confidence intervals (shaded area) are shown. The reference serum albumin level was set at 4.0 g/dL

**Fig. 5:**
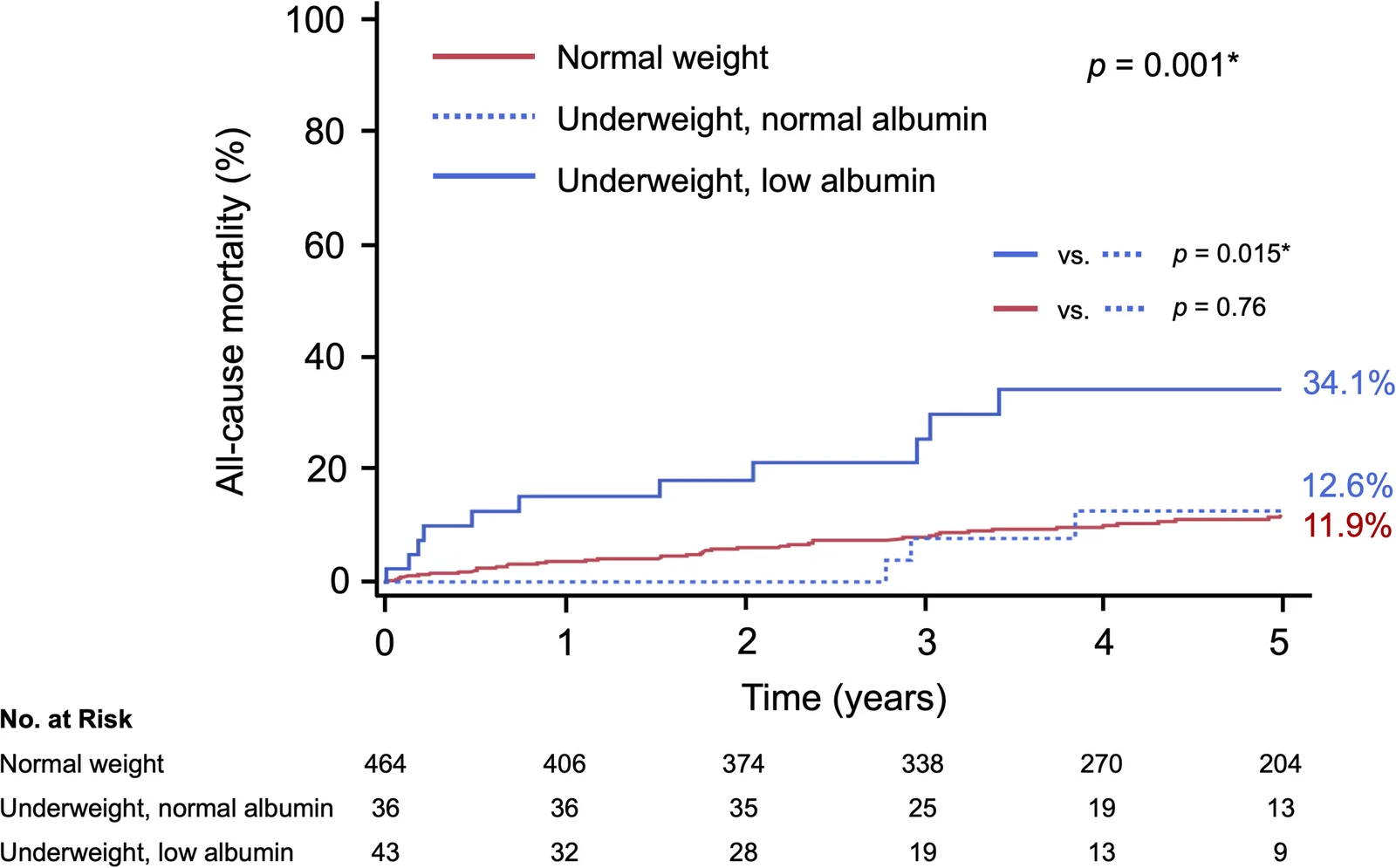
Kaplan–Meier curves for all-cause mortality comparing normal-weight patients, underweight patients with low serum albumin (< 4.0 g/dL), and underweight patients with normal serum albumin (≥ 4.0 g/dL)

Among underweight patients, the median preoperative serum albumin level was 4.0 g/dL. Restricted cubic spline analysis suggested a continuous inverse association between serum albumin and the hazard of all-cause mortality (Fig. 4). In Cox proportional hazards analyses, serum albumin was significantly associated with all-cause mortality in the univariable model (HR: 0.16; 95% CI: 0.04 to 0.60; *p* = 0.006). Serum albumin remained independently associated with all-cause mortality in both the minimally adjusted model (Model 1; adjusted HR: 0.13; 95% CI: 0.03 to 0.51; *p* = 0.005) and the more fully adjusted model (Model 2; adjusted HR: 0.15; 95% CI: 0.03 to 0.69; *p* = 0.014) (Table 4). Underweight patients were additionally stratified by the median albumin level into lower albumin (< 4.0 g/dL, *n* = 43) and normal albumin (≥ 4.0 g/dL, *n* = 36) subgroups. The low albumin subgroup had a higher 5-year all-cause mortality than the normal albumin subgroup (34.1% vs. 12.6%, log-rank *p* = 0.015). No significant difference in 5-year all-cause mortality was observed between underweight patients with normal albumin and normal-weight patients (12.6% vs. 11.9%, log-rank *p* = 0.76) (Fig. 5).

**Table 4:** Preoperative serum albumin and all-cause mortality in underweight patients: multivariable Cox analysis

	Univariable		Multivariable (Model 1)		Multivariable (Model 2)	
	HR (95% CI)	*p* value	Adjusted HR (95% CI)	*p* value	Adjusted HR (95% CI)	*p* value
Albumin, per 1.0 g/dL	0.16 (0.04–0.60)	**0.006**	0.13 (0.03–0.51)	**0.005**	0.15 (0.03–0.69)	**0.014**
Age, per 1 year	1.02 (0.95–1.11)	0.63	1.03 (0.95–1.13)	0.45	1.05 (0.96–1.17)	0.27
Female	1.17 (0.39–3.49)	0.78	1.64 (0.53–5.10)	0.39	1.60 (0.51-5.00)	0.42
BMI, per 1.0 kg/m^2^	0.77 (0.56–1.11)	0.16			0.66 (0.43–1.02)	0.060
Chronic kidney disease	7.47 (1.66–33.57)	**0.009**			5.84 (1.20-28.32)	**0.029**
LVEF, per 1%	0.99 (0.96–1.03)	0.50			1.02 (0.98–1.06)	0.44

Model 1 was adjusted for age and sex. Model 2 was adjusted for age, sex, BMI, chronic kidney disease, and LVEF.

Bold indicates *p* < 0.05. BMI, body mass index; CI, confidence interval; HR, hazard ratio; LVEF, left ventricular ejection fraction.

## Discussion

The main findings of this study are summarized as follows: (1) Underweight patients had highest all-cause mortality: (2) Lower BMI was identified as an independent predictor of all-cause mortality: (3) Among underweight subgroup, lower serum albumin was independently associated with a higher risk of all-cause mortality.

BMI is widely recognized as a fundamental indicator of nutritional assessment for evaluating malnutrition risk, and underweight is considered a form of malnutrition, with a BMI < 18.5 kg/m² commonly used as the threshold [7–9]. Large epidemiological studies, including the Japanese pooled cohort and the contemporary U.S. National Health Interview Survey, have both demonstrated a J-shaped association between BMI and all-cause mortality, with increased risk not only with higher BMI but within the underweight range [10, 11] In patients with chronic heart failure, underweight or low BMI was independently associated with a higher risk of all-cause mortality, as reported in the CHARM program analysis [12]. Several studies suggested that underweight patients experience higher postoperative mortality after cardiac surgery [13–19]. However, these studies were primarily conducted in Europe and North America, where the proportion of underweight patients was extremely low, approximately 1% for those with a BMI < 18.5 kg/m², [17,18] and 1.6–5.6% for those with a BMI < 20 kg/m² [13, 14, 16, 19]. In contrast, in our study, 10.2% of patients were classified as underweight (BMI < 18.5 kg/m²), which is roughly tenfold higher than the rate reported in Western populations. Accordingly, the clinical significance of underweight may also differ from that in Western populations.

In patients undergoing TAVR, the clinical impact of underweight has been examined in several studies. The proportion of underweight patients (BMI < 18.5 kg/m²) was reported to be 1.7–2.6% in Western populations, [20–22] whereas it was markedly higher, at 14.3–15.1% in Japanese cohorts [1,23, 24]. All of these studies consistently demonstrated that underweight status was associated with increased mortality, [1, 6, 20–23] and several further suggested that an excess of non-cardiovascular deaths may have contributed to this increased risk [23, 24]. Similarly, in the present study, underweight was associated with approximately threefold higher mortality after SAVR compared with normal-weight patients. Moreover, this excess mortality appeared to result primarily from a higher incidence of non-cardiovascular death. In particular, infection-related death was more frequent among underweight patients, suggesting that impaired nutritional status and frailty may have contributed.

However, some underweight Japanese patients are not frail or malnourished but are constitutionally small and healthy, and such patients may not be at high risk for SAVR. In a further analysis among underweight patients, this study showed that serum albumin level effectively stratified their prognosis. Serum Albumin is the main blood protein that maintains fluid balance, transports substances, and protects against inflammation and clotting, and low levels are often caused by poor nutrition or chronic inflammation [25]. Previous studies have highlighted the prognostic importance of nutritional status in patients undergoing cardiovascular surgery. Lee et al. reported that the prognostic nutritional index, which incorporates serum albumin, was useful for predicting early clinical outcomes after adult cardiac surgery [26]. In addition, Hayashi et al. showed that a low prognostic nutritional index was associated with postoperative complications and worse survival in patients undergoing cardiovascular surgery, including an Asian population similar to that in the present study [27]. Although these studies evaluated the prognostic nutritional index rather than the isolated effect of serum albumin, they support the clinical importance of preoperative nutritional assessment in cardiovascular surgery. Our findings suggest that serum albumin alone may also be useful for risk stratification in underweight Japanese patients undergoing SAVR. Although a serum albumin level of 3.5 g/dL is commonly used as the cutoff for hypoalbuminemia, higher thresholds have also been applied [25, 28]. In the present study, we used the median serum albumin level of 4.0 g/dL as the cutoff to stratify underweight patients. Even among underweight patients, those with preserved serum albumin levels (≥ 4.0 g/dL) exhibited favorable survival outcomes. These findings indicate that a subset of underweight Japanese patients experience favorable outcomes after SAVR. Incorporating serum albumin into preoperative risk assessment could help identify underweight patients most likely to benefit from SAVR. In contrast, underweight patients with low serum albumin showed significantly higher mortality, indicating a particularly high-risk subgroup. In such patients, less invasive approaches, including TAVR and minimally invasive surgical AVR, may be considered depending on overall risk profile, anatomy, and the treating center’s experience [12]. Overall, our findings suggest that serum albumin measurement provides a simple and effective tool for risk stratification in underweight patients undergoing SAVR. Therefore, in underweight patients with severe AS, comprehensive risk evaluation incorporating serum albumin should be considered when determining the optimal treatment strategy.

### Study limitation

This study has several limitations. First, it used a retrospective design and is therefore subject to selection bias. Second, the study population consisted only of Japanese patients with relatively small body size, and potential racial or ethnic differences were not assessed. Therefore, the generalizability of these findings to other populations with larger average body sizes may be limited. Third, data regarding body-weight changes within the six months prior to surgery were unavailable. Although weight loss represents an important criterion for nutritional assessment, it is often based on patient self-report, making it difficult to obtain objective documentation. Fourth, BMI reflects overall body size but does not capture body composition, such as muscle mass. Although serum albumin was a useful prognostic marker in our analyses, it can be influenced by fluid balance and acute inflammatory states, including infection. Therefore, BMI and serum albumin should be interpreted cautiously as indicators of nutritional status or frailty. Fifth, the number of events in the underweight subgroup was limited, and therefore the multivariable analyses may still have been affected by overfitting despite careful restriction of the number of covariates.

## Conclusion

Underweight was associated with higher mortality after SAVR. Serum albumin provided additional risk stratification within the underweight group, with preserved albumin associated with more favorable outcomes and low albumin identifying a particularly high-risk subgroup.

## Supplementary Information

Below is the link to the electronic supplementary material.

Supplementary Material 1

## Abbreviations

BMI: Body mass index
SAVR: Surgical aortic valve replacement
AS: Aortic stenosis
TAVR: Transcatheter aortic valve replacement
STS-PROM: The Society of Thoracic Surgeons Predicted Risk of Mortality
IQR: Interquartile range
