# Supplementary material for: Prognostic utility of serum albumin in underweight patients who underwent isolated surgical aortic valve replacement
Source: Gen Thorac Cardiovasc Surg. 2026 Apr 27;74(10):939–49. doi: 10.1007/s11748-026-02299-3 (PMC13633306; doi:10.1007/s11748-026-02299-3)
Supplement: Supplementary file 1 [file 11748_2026_2299_MOESM1_ESM.docx]

Supplemental Figure E1. The distribution of causes of non-cardiovascular death according to body mass index categories.
